# Evolving Techniques in Pancreatic Surgery

**DOI:** 10.1155/2016/4289724

**Published:** 2016-03-28

**Authors:** Dejan Radenkovic, Michael B. Farnell, Claudio Bassi, Marc Besselink

**Affiliations:** ^1^Clinic for Digestive Surgery, Clinical Center of Serbia and School of Medicine, University of Belgrade, 11000 Belgrade, Serbia; ^2^Department of Surgery, Division of Subspecialty General Surgery, Mayo Clinic, 200 First Street SW, Rochester, MN 55905, USA; ^3^Unit of General Surgery B, The Pancreas Institute, University of Verona Hospital Trust, Verona, Italy; ^4^Department of Surgery, Academic Medical Center, Amsterdam, Netherlands

Diseases of the pancreas and periampullary region form an important clinical group of malignant and also benign diseases which still carry relatively high morbidity and mortality rates. Pancreatic ductal adenocarcinoma, in particular, is associated with very poor outcome as the number of new cases per year is just slightly higher than the number of deaths from this disease. Therefore, treatment of these conditions should always be based on both the highest level of evidence and technical expertise.

Surgery for diseases of the pancreas and periampullary region has evolved substantially during the last 15 years and several new surgical techniques have been described. According to recent randomized trials and advances in medical treatment, several surgical dogmas have been refuted.

The majority of patients with pancreatic and periampullary cancer at the time of diagnosis have advanced disease. However, in specialized centers, 10–15% of the patients with these malignances are suitable for resection. In these centers, the morbidity and mortality associated with major pancreatic resection have been reduced considerably in recent years. It is an undisputed fact that pancreatic resection ranks as one of the most, if not the most, complicated and technically challenging surgical procedures. It not only is a demanding technical exercise but also exerts a substantial logistical strain on healthcare resources.

The group from Heidelberg addressed the importance of vascular resections during pancreatic surgery. The limits of resection in patients with pancreatic and periampullary cancers have been extended. The recently published consensus paper by the International Study Group for Pancreatic Surgery (ISGPS) provided up-to-date definitions of borderline and extended resections. These definitions help clinicians worldwide to apply extended surgical approaches to an increasing number of surgical candidates. These procedures include vascular and multivisceral resections.

When dictated by tumor involvement, resection of portal and superior mesenteric and splenic veins should be performed routinely in patients if the patient's general condition allows and there are no major comorbidities. The decision for arterial resections should be strictly individualized. This type of surgery should be reserved for fit, younger patients, with preoperative careful examination of all aspects of this extended surgery. The majority of patients with infiltration of coeliac trunk or superior mesenteric artery should be considered for neoadjuvant therapy and then reevaluated for postponed surgical intervention and resection of infiltrated vessels.

D. Hartmann and H. Friess addressed the current status of the role of the surgery in the treatment of chronic pancreatitis. Surgical intervention in patients with chronic pancreatitis is mainly performed to resolve the complications of disease. In the majority of patients, intractable pain is the major indication for surgery, although jaundice, duodenal obstruction, and portal vein thrombosis are not rare. The goal of the surgery is pain reduction and management of complications, while preserving exocrine and endocrine pancreatic function and improving quality of life. Timing of surgery is an important feature during the prolonged natural history of the disease. New findings support early surgery for pain management in chronic pancreatitis patients, since early surgical intervention is associated with an improved postoperative pain relief, a reduced risk of pancreatic insufficiency, and decreased reintervention rates in comparison to conservative step-up approaches.

Duodenum-preserving pancreatic head resections (including Beger, Frey, and Berne procedures) seem to be superior in peri- and postoperative outcome parameters and quality of life compared to partial pancreatoduodenectomy (Whipple procedure) while being equally effective in postoperative pain relief, overall health, and postoperative endocrine sufficiency.

Organ-sparing operation at the right point of time carries a better outcome than performing an extended resection as a last resort once all therapeutic options are exhausted. An early timing of surgical therapy is crucial for the outcome of patients with painful CP and the indication of surgery should be considered early once symptoms are unambiguous.

The group from Verona evaluated technical aspects, indications, and results of the application of Radiofrequency Ablation (RFA) and Irreversible Electroporation (IRE) on locally advanced pancreatic cancer (LAPC).

RFA and IRE are the most frequently applied ablative techniques for the management of locally advanced pancreatic cancer. These techniques are versatile, since they can be performed at laparostomy, or via laparoscopic, endoscopic, or percutaneous routes. It has been demonstrated that their use is safe; however, since serious adverse events can occur, they are best performed at high volume centers and by high volume surgeons.

The application of RFA and IRE is recommended in a multidisciplinary decision-making setting and they should be used especially on locally advanced tumors that have not demonstrated a propensity to metastasize. That said, properly designed studies are still needed to assess their efficacy.

Finally, one of the most intriguing aspects of the application of RFA and IRE is their presumed stimulation of the immune system against the cancer. Once this aspect is further clarified and possibly confirmed, their use within the natural history of pancreatic cancer will become clearer. The group from Glasgow described the treatment of infected necrotizing pancreatitis. Treatment of acute pancreatitis is currently based on the definitions set forth in the 2012 Revised Atlanta Classification. Since the PANTER trial, there is now consensus that a “step-up approach” starting with catheter drainage and, if needed, followed by minimally invasive necrosectomy is the preferred treatment approach to infected necrotizing pancreatitis. The most commonly used minimally invasive strategies for necrosectomy are transgastric necrosectomy, percutaneous necrosectomy, and video-assisted retroperitoneal debridement (VARD). The recently completed TENSION trial compared transgastric necrosectomy with VARD within a “step-up approach,” but the final results are awaited. The advantage of transgastric necrosectomy is that it minimizes the risk of a pancreatocutaneous fistula which can be very problematic in case of “central gland necrosis.” The major disadvantage of transgastric necrosectomy is that multiple, often lengthy, procedures are required, whereas, with VARD via a 3–5 cm incision, only 1-2 procedures are sufficient in the majority of patients. Percutaneous necrosectomy probably holds the middle ground between these two options and several large series have described good outcomes.

The group from Belgrade overviewed the role of interventional treatment of abdominal compartment syndrome (ACS) during severe acute pancreatitis (SAP). Some unresolved questions persist which include the role of medical treatment and the indications for and the timing of interventional techniques. Currently, there is no unanimity of opinion regarding surgical or other interventional treatments for ACS during the course of SAP.

Critically ill patients with acute pancreatitis have a considerable risk for developing intra-abdominal hypertension. Routine measurement of intra-abdominal pressure is recommended, allowing the identification of patients at risk of abdominal compartment syndrome. First line therapy for this life-threatening complication is conservative treatment aiming to decrease IAP and to restore organ dysfunction. If nonoperative measures are not effective, early abdominal decompression is mandatory. Percutaneous catheter drainage should be the first step in interventional treatment which can relieve ACS. When ACS persists, surgery is indicated.

Timing of abdominal decompression also remains uncertain in the treatment of these patients. However, the Finnish group published their experience with 26 patients regarding early and late decompression. Results clearly showed that early decompression (first 4 days) was associated with a significantly lower mortality rate, compared with late decompression (after 4 days).

Midline laparostomy seems to be the method of choice, although full-thickness transverse subcostal bilateral laparostomy, subcutaneous linea alba fasciotomy, and fasciotomy of the anterior rectus abdominis sheath have been described as alternative options. Since laparostomy carries significant morbidity, randomized studies are needed to establish firm advantages over other techniques.

Better understanding of pathophysiology of diseases of the pancreas and periampullary region has led surgeons to change some traditional surgical approaches and develop new innovative techniques. Technological advances which have improved the safety and efficacy of pancreatic surgery have been performed mainly in selected high volume centers. In order to improve both quality of life and duration of survival for our patients, novel surgical techniques should become more widely available and accepted. Studies with sound design and statistical methodology are urgently needed.

In the present special issue, the authors have addressed new understanding of the pathophysiology of diseases of the periampullary region and pancreas. They have focused their interest in evidence-based, novel surgical techniques and approaches and challenging clinical settings have been emphasized.

*Dejan Radenkovic* *Dejan Radenkovic* *Michael B. Farnell* *Michael B. Farnell* *Claudio Bassi* *Claudio Bassi* *Marc Besselink* *Marc Besselink*

